# Tusks, testosterone and personality in male Asian elephants (*Elephas maximus*)

**DOI:** 10.1098/rsos.250490

**Published:** 2025-08-27

**Authors:** Hoedric Huguet, Martin W. Seltmann, Win Htut, Michael Briga, Carly Lynsdale, Virpi Lummaa

**Affiliations:** ^1^ Department of Biology, University of Turku, Turku, Finland; ^2^ Myanma Timber Enterprise, Yangoon, Myanmar; ^3^ Turku Collegium for Science, Medicine and Technology, University of Turku, Turku, Finland; ^4^ University of Helsinki, Helsinki, Finland

**Keywords:** androgens, hormones, behaviour, defence, ornament, sexual selection, proboscidean, evolution, phenotypic trait, long-lived species

## Abstract

Male Asian elephants exhibit phenotypic diversity in tusk development, with long, short and tuskless bulls varying in frequency among different populations. Although the factors that maintain tusk variation in Asian elephants remain unclear, tusks are considered a secondary sexual characteristic probably influenced by sexual selection. In this study, we examined the relationship between tusk diversity, faecal testosterone metabolite (FTM) and personality in male Asian elephants aged 5–60 years living in semi-captive conditions within their native habitat in Myanmar. Males with different tusk types did not display differences in FTM levels or in scores for the three main personality factors, but there were some distinctions in the trait loadings within each factor: attentiveness, activity and dominance loaded more strongly for long-tusk males, while traits like obedience, slowness and aggression showed stronger associations in short-tusk males. Our study suggests that the differences between long- and short-tusk males in testosterone levels and personality traits were, respectively, negligible and nuanced, emphasizing the complexity of tusk expression and evolution in Asian elephants.

## 1. Introduction

Many sexually reproductive species develop secondary sexual characteristics (SSCs) that enhance mating opportunities. These SSCs often differ between the sexes, including exaggerated coloration, behaviours or ornamentation [1]. Examples include body size differences between male and female mammals [2,3], antlers in deer [4], tusks in narwhals (*Monodon monoceros*) [5] and in Asian elephants (*Elephas maximus*) [6].

**Figure 1.**
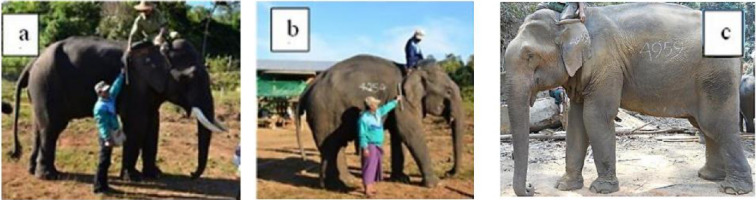
Phenotypic variation in tusk expression in the study population: pictures from Myanmar timber elephants: (a) a 45-year-old long-tusk male; (b) a 42-year-old short-tusk male; and (c) a 37-year-old tuskless male.

Our understanding of tusks and the variation in tusk size in Asian elephants remains poor. Unlike African elephants (*Loxodonta africana*), where both sexes develop tusks, Asian elephants primarily exhibit tusks in males [6,7]. Asian elephants display three distinct tusk types, with some males possessing long tusks, others short tusks and some lacking tusks entirely (figure 1) [8,9]. In Asian elephants, tusks begin to emerge by the age of five years, with long tusks continuing to grow until approximately 30 years old, resulting in inter-individual variability in tusk length, with measurements ranging from about 50 to 180 cm among similarly aged individuals (Mudumalai Wildlife Sanctuary, India) [10,11]. The ecological, physiological and evolutionary basis of the variation in tusk morphology remains poorly understood.

The Fisherian runaway model posits coevolution between female preferences and male SSCs, with females favouring increasingly exaggerated male features [12]. This model suggests that the production of these SSCs primarily occurs in high-quality males who can afford the physiological costs, which are offset by the mating advantages they provide [13–16]. For instance, antlers regrow annually for the rutting season, require considerable energetic investment [17,18] and serve as indicators of male fitness [4,19,20], to establish social dominance and secure access to females in oestrus [21]. Elephant tusks are probably subject to some form of sexual selection as well, particularly given that tusks have low tensile strength and are unlikely to serve a functional role as tools or weaponry [22]. For example, when tusked and tuskless males are equal in body size and musth status, tusked males are more likely to gain access to females [22]. Hence, although tusks in Asian elephants are probably SSCs, we know almost nothing about the biological underpinnings of variation in tusk morphology.

In many species, it has been observed that SSCs and female preference can be influenced by male testosterone levels (dark-eyed junco, *Junco hyemalis* [23]; superb fairy-wren, *Malurus cyaneus* [24]; *E. maximus* [7,25,26]). While testosterone–SSC size correlations have been extensively researched during the mating season, studies on this relationship outside the reproductive period remain limited [27,28]. In the Iberian ibex (*Capra pyrenaica hispanica*) and European mouflon (*Ovis musimon*), horn growth occurs during sexually inactive periods, with low testosterone levels promoting horn growth and high levels suppressing it during the rutting season [29–31]. A similar mechanism is suggested for antler development in deer [32,33].

In the Myanmar population of Asian elephants, veterinarians differentiate three male tusk types (figure 1). Asian elephant tusks grow asymptotically, starting when elephants are calves until the age of 30 years, marking the beginning of the slowdown in growth [11]. This suggests that the conditions determining tusk phenotypes operate largely independently of the testosterone surges occurring during adult musth, i.e. an annually recurring male reproductive period characterized by a sharp rise in hormone secretions and aggressive behaviour, typically lasting between a few days and several months [34–37]. Interestingly, the relationship between testosterone levels during non-musth periods—the most substantial part of the life cycle—and tusk length remains unclear. Understanding these mechanisms is essential for unravelling the hormonal factors that drive tusk variation and can provide broader insights into the development of sexual dimorphism in elephants.

In addition to androgens such as testosterone, behavioural consistency, or personality (e.g. dominance, aggression or sociability), is another trait that may be linked to SSCs [38–40]. For example, in mandrills, dominant males display bright facial coloration and heightened aggression, which decrease with social rank [41]. In red deer and bighorn sheep, larger antlers or horns correlate with bold, aggressive and dominant behaviours, enhancing mating opportunities through status and contest success [42,43]. These examples support the idea that SSCs and personality traits co-evolve as integrated components of reproductive strategies shaped by ecological and hormonal constraints, which offers insight into the evolution of reproductive strategies, behavioural and phenotypic diversity within one sex. The association between SSCs and personality can emerge indirectly owing to androgens’ influence on aggressive behaviours [44]. In Asian elephants from Myanmar, males exhibit higher aggression than females [45,46], a difference that is often associated with elevated testosterone levels during musth [25]. If male personality outside musth is directly driven by their testosterone concentration, and if long-tusk males have lower testosterone concentration than their short-tusk peers, we expect long-tusk males to show less aggression than short-tusk males.

This study is, to our knowledge, the first to investigate the associations between tusk type, testosterone concentration and personality traits in semi-captive Asian elephants living in their natural habitat. We use data from semi-captive male Asian elephants in Myanmar, including 6 years of faecal testosterone metabolite (FTM) samples and 5 years of personality ratings, collected outside of musth. We formulated two hypotheses. First, given that most of the tusk growth may occur outside musth and because examples in the ibex and mouflon showed more horn growth when testosterone levels are low [29–31], we expect that long-tusk males would exhibit lower testosterone levels than their short-tusked peers. Second, because testosterone is often linked to a more aggressive personality, we expect long-tusk males to have less aggressive and more social personalities than short-tusk males.

## 2. Material and methods

### 2.1. Study animals

We studied individuals that are part of a population of semi-captive elephants in Myanmar, owned by the government and the Myanmar Timber Enterprise (MTE) [47,48]. All individuals are located in three areas of the Sagaing region in northern Myanmar: East-Katha and West-Katha (24°10′ N, 96°19′ E) and Kawlin (23°46′ N, 95°40′ E). Elephants in this semi-captive population work during the day and can roam freely in the forest at night, foraging, interacting with conspecifics and mating with wild and other semi-captive elephants. The reproduction of working elephants occurs without supervision, and their diet is not supplemented, which means that their foraging and breeding patterns, physical condition and vital rates are comparable to those of wild Asian elephants [49]. They work pulling and pushing logs during the cold (October–January) and monsoon (June–September) seasons and rest during the hot season (February–May). Most of the elephants included in the study were born in semi-captivity (FTM: *n* = 72 of 80 individuals or 89%; personality: *n* = 81 of 105 individuals or 88%), while eight individuals for FTM and 24 individuals for personality tests originated from the wild. All wild-caught animals were tamed before 1997, which marks the prohibition of wild capture [47,50]. Elephants are managed daily during working hours (5–8 h d^−1^, 5 d wk^−1^) by their head rider (mahout) in logging regions, where they handle and pull logs [50,51]. MTE veterinarians provide fundamental care, such as treating wounds and injuries sustained during work. The working tasks of the timber elephants vary according to age: calves are kept with their mothers and allomothers, who nurse them until the age of 4 or 5 years [48,52], followed by approximately one month at a designated taming camp [53,54]. After taming, the elephants are trained to follow their mahouts’ commands and to perform different tasks. Until they are around 20 years old, juvenile elephants are used as light-duty transport animals. After that, elephants also begin to work as draft animals, pulling and handling logs and transporting them out of the forest until their retirement at around age 54 [51]. Retired elephants are monitored and cared for by their mahouts and MTE veterinarians until death. MTE elephants are never culled.

Every individual is assigned a unique identification number (ID) and is subject to individual follow-up throughout their life, recorded in personal logbooks by the veterinarian responsible for their health monitoring. This information includes details about birth, calving, maternal identity, sex, tusk type (long, short or tuskless), health status, physiological and morphological data and dates of death. Male tusks are classified at the age of 4–5 years by MTE veterinarians into tuskless (no visible tusks or vestigial tushes), short-tusk (which do not grow to exceed 20–30 cm in length in a lifetime) or long-tusk (which grow continuously throughout a male’s lifetime). We concentrated on individuals with short and long tusks, as tuskless males are uncommon in our study population, which reflects the proportional representation of tuskless males within Myanmar and the surrounding region.

### 2.2. Collection of faecal testosterone samples

The FTM were measured from 1852 faecal samples of 80 male Asian elephants, ranging from 5 to 64 years old. Testosterone levels can vary with age [55,56], but the median ages for both tusk types of males are almost identical (long-tusk: median = 10.19, s.d. = 15.93, 95% confidence interval (CI) = (5.20; 59.55); short-tusk: median = 10.63, s.d. = 14.25, 95% CI = (5.63; 59.52); table 1).

**Table 1.** Descriptive statistics for FTM concentrations and age in long-tusk (*n* = 58) and short-tusk (*n* = 22) Asian elephant males sampled in this study, reported in ng g^−1^ of dried faeces. (Sample size (*N*_obs_) indicates the number of samples collected for each tusk type. The number of individuals (*N*_ID_) indicates the number of males for each tusk type. s.d., standard deviation.)

FTM							age			
	*N*_obs_	*N*_ID_	mean	min.	max.	s.d.	med.	min.	max.	s.d.
long-tusk	1346	58	49.99	3.24	477.36	48.75	10.19	5.00	64.34	15.93
short-tusk	524	22	46.68	5.19	416.68	42.71	10.63	5.00	60.87	14.25

Faecal samples were collected immediately after defecation for each individual on a regular, almost monthly basis, from 2013 to 2020. From boluses of faecal material, 4.5 g of sample was extracted from both the centre and the edge, as described in Lynsdale *et al*. [56,57]. The measurements of samples taken from individuals undergoing taming were excluded because taming is associated with a six-month-long increase in stress [53]. The samples were transferred to a cooler box for up to 8 h before being stored in a freezer at −20°C. Sample analysis was conducted between 2016 and 2021, at the Veterinary Diagnostic Laboratory of Chiang Mai University, Thailand, in 12 separate batches. The faecal samples were dried in a hot air oven at 50°C. Then, an extraction step for FTM was carried out following the boiling extraction protocol [57].

### 2.3. Testosterone enzyme immunoassays

A non-invasive hormone analysis was employed to measure testosterone levels from faecal samples. FTM concentrations were quantified via enzyme immunoassay validated for elephants [58]. The dilution of the FTM samples was 1 : 4. Ninety-six-well plates were pre-coated with 150 μl (10 μl ml^−1^) coating antibody (goat anti-rabbit IgG) diluted in a coating buffer, then stacked and left for 24 h at room temperature. The coating buffer was subsequently removed from each plate, and 0.25 ml of blocking buffer was dispensed into each well. Blocked plates were stacked and left at room temperature for 4–24 h. The wells were then emptied and blotted dry, and the plates were transferred to a desiccator, where they remained at room temperature until the inside humidity fell below 20%. The dried plates were individually packaged and heat-sealed inside labelled foil ziplock bags before being stored at 4°C until needed. Pre-coated goat anti-rabbit plates were used. In total, 75 μl of assay buffer was added to the non-specific binding (NSB) wells, and 50 μl of assay buffer was added to the wells designated as maximum binding wells (0 pg ml^−1^). Next, 50 μl of standard controls and samples were introduced into the wells of the plates. Horseradish peroxidase-conjugated testosterone tracer with a concentration of 1 : 20 000 was immediately added to each well, followed by 25 μl of testosterone polyclonal antibody (R156/7) with a concentration of 1 : 1 10 000 added to all wells except the NSB wells. The plate was then covered with a plate sealer and shaken at room temperature for 2 h. Each well was washed four times with a wash buffer (1 : 20 dilution), and 100 μl of 3,3',5,5'-tetramethylbenzidine substrate solution was added to each well before incubation at room temperature for approximately 30 min without shaking. Finally, 50 μl of stop solution was added to each well, and the optical density generated from each well was read at 450 nm using a microplate reader. The assay had a sensitivity of 0.0567 ng ml^−1^, with intra-assay coefficients of variation (CV) below 10 and inter-assay CV of 12.73. FTM concentrations were expressed as nanograms per gram of dry faecal matter (ng g^−1^). The testosterone range values in the dataset start from 3 ng g^−1^ at the minimum to 477 ng g^−1^ of faeces.

### 2.4. Collection of personality data

To quantify the elephants’ personality types, we used the personality data and model from a previous study [40], where elephant personality was assessed through a questionnaire completed by mahouts familiar with each focal elephant. Each observation in our dataset corresponds to one elephant personality evaluation collected between 2014 and 2018, for a total of 149 observations, of which 109 are on 80 long-tusk individuals and 40 are on 25 short-tusk individuals. Some individuals were evaluated multiple times across different years [50]. Each observation was performed by one to three raters, with a total of 155 raters, and all raters were either the elephant’s own mahout or the head mahout from the same working group, ensuring substantial familiarity with the elephants. For the elephants’ own mahouts, the median raters’ age was 24 years (interquartile range: 19−32 years; range: 15−59 years), and the median rater experience with their elephant was 2 years (interquartile range: 1−4 years; range: 1 day−22 years). Mahouts judged the frequency of 28 personality items, following the same validated protocol described in [40], using a four-point scale, where 1 signifies ‘very rarely’, 2 ‘occasionally’, 3 ‘quite a lot’ and 4 ‘most of the time’. To ensure independence, mahouts were instructed not to discuss their ratings. Following exploratory factor analysis (EFA), 15 personality items were retained, loading onto three factors (attentiveness, sociability, aggressiveness; see the electronic supplementary material, figure S1). The inter-rater reliability estimates of these 15 items were almost the same as those in [40], with a mean Fleiss’ kappa value of 0.186 and a range between 0.132 (for affectionate) and 0.375 (for dominant). The inter-rater reliability indicates a moderate repeatability of elephant personality over time, with the variation over time possibly owing to changes with age, mahouts or elephant personality.

### 2.5. Statistical analyses

#### 2.5.1. Faecal testosterone metabolites analysis

All analyses were conducted using the software R (v. 4.3.2; R Core Team, 2023). For all FTM analyses, we employed general linear models with the function ‘glmmTMB’ v. 1.1.7 from the package ‘TMB’ [59] and log-transformed FTM to meet the homoscedasticity of residuals requirement. Besides our main variable of interest, tusk type (two levels: long-tusk, short-tusk), we incorporated the following three covariates as fixed effects: (i) sample collection season (three levels: monsoon, hot and cool season); (ii) region (three levels: East-Katha, West-Katha and Kawlin); and (iii) age (continuous variable) since these factors are known to influence FTM concentrations [60,61]. We used the likelihood ratio test to assess the statistical significance of the fixed effects. Furthermore, we accounted for the non-independence of repeated measures by including two random terms: the elephant’s individual identity and the batch year of faecal sample extraction. To visualize the model results, we extracted the means of predicted values for each tusk group, obtained using the function ‘predict’ and type ‘response’.

#### 2.5.2. Personality analysis

We analysed elephant personality using two complementary approaches on each tusk type. First, we used confirmatory factor analysis (CFA) to validate the good-fitting model [40,45] for each tusk type. Second, we used EFA to assess whether tusk types differ *within* the three main personality factors.

##### 2.5.2.1. Confirmatory factor analysis

To compare the personalities of short- and long-tusk elephants, we used the three-factor personality model previously identified in this population [40,45]. We conducted an initial verification step and tested whether our data fitted this three-factor model by examining, for each tusk type, the following four fit indices: the comparative fit index (CFI), the Tucker–Lewis index (TLI), the standardized root mean square residual (SRMR) and the root mean square error of approximation (RMSEA). The CFI and TLI are fit indices, where a value of 1 indicates a perfect fit of our data, and values above 0.90 correspond to an acceptable fit. The RMSEA and SRMR indices reflect a good fit when the estimate is smaller than 0.08 [62].

Next, we tested for measurement invariance between short- and long-tusk males. Measurement invariance assesses the similarity of personality factor structure between groups (here tusk type). If measurement invariance is confirmed, then the personality factors possess the same meaning across groups, allowing for comparing their factor means and variances. To test for measurement invariance, we estimated and compared increasingly constrained CFA models. The first model is a configural invariance model, which is an unconstrained model. Next, we examined the metric and scalar invariances. Metric invariance is a constrained version of the configural model where the factor loadings are expected to be equal across groups while the intercepts are permitted to vary between groups. Metric invariance facilitates the comparison of factor variances and covariances between these groups. Scalar invariance, in turn, is a constrained version of the metric invariance model, where both the factor loadings and intercepts are assumed to be equal across groups. Scalar invariance enables the comparison of factor means between the groups. Scalar invariance was tested using a chi-square difference test.

##### 2.5.2.2. Exploratory factor analysis

Next, we conducted a more detailed analysis of the potential personality differences between males with different tusk types using EFA (with the ‘oblimin’ rotation to allow for correlation between factors) by comparing the 15 personality items underlying the three aforementioned personality factors.

To test the sampling adequacy of the correlation matrix (function ‘cor’) for data subsets (i.e. long- and short-tusk males), we performed the Bartlett sphericity test (function ‘cortest.bartlett’) and the Kaiser–Meyer–Olkin (KMO; function ‘KMO’) measure of sampling adequacy (all functions from the ‘psych’ package [63]) on each subset. For both data subsets, the Bartlett sphericity test was significant (*p* < 0.01), indicating that the correlation matrix was significantly different from a matrix in which traits were not correlated, thereby supporting further analyses [64]. Furthermore, the overall KMO measure of sampling adequacy was 0.92 and 0.86 for long- and short-tusk males, respectively, exceeding the threshold value of 0.7, thus also fulfilling the criterion for the matrix being suitable for further analyses (see [65] and references therein). To estimate the number of factors to retain in the factor solution, we combined three approaches [65]: (i) eigenvalues >1; (ii) scree plot analysis; and (iii) Horn’s parallel analysis with 1000 iterations (package ‘paran’ [66]). We found evidence supporting the retention of three factors for long-tusk males, while for short-tusk males, the three approaches (eigenvalues, scree plot analysis and Horn’s parallel analysis) retained between 1 and 3 factors (electronic supplementary material, table S1). As a one-factor solution is not interpretable and because we wished to ensure comparability of personality structure between both tusk types, we decided to proceed with three factors for both tusk types. Once we determined the number of factors to retain for each subset, we performed EFA using the ‘psych’ package (all functions [63]) and the ‘fa.sort’ function from ‘fungible’ [67] for the two data subsets on elephants with short and long tusks.

## 3. Results

### 3.1. Faecal testosterone metabolites

**Figure 2.**
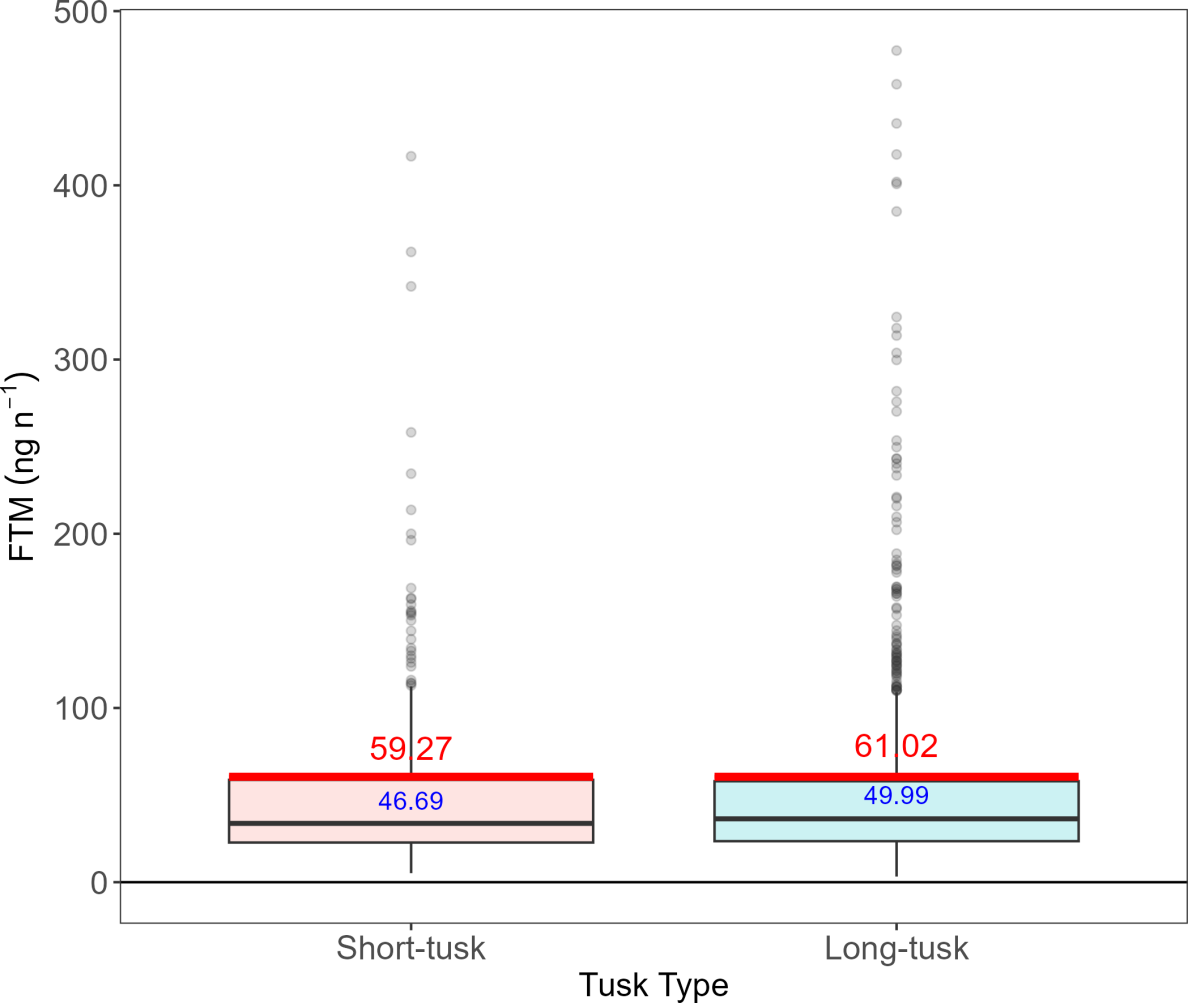
FTM concentrations (ng g^−1^) in male Asian elephants (*n* = 80 individuals) with short (*n* = 22 individuals) and long (*n* = 58 individuals) tusks. Box plots and blue numbers show FTM means from the raw data for each tusk type. The red lines and numbers indicate the mean predicted testosterone values from the final model in table 2.

Descriptive statistics indicate that long-tusk males have slightly higher FTM concentrations than short-tusk males (mean_Long-tusk_ = 49.99 ng g^−1^ and mean_Short-tusk_ = 46.68 ng g^−1^; table 1). The standard deviations (s.d.) for both tusk types are similar and overlap with each other’s means: 42.71 ng g^−1^ for short-tusk males and 48.74 ng g^−1^ for long-tusk males (figure 2). Consistent with this observation, a statistical model that accounts for the repeated measures and covariates demonstrates that the difference in FTM between tusk types is not statistically significant (figure 2; *χ*^2^ = 1.08, *p* = 0.30; table 2). FTM concentrations increase with age (*χ*^2^ = 7.78, *p* = 0.005; table 2), but the interaction between age and tusk type is not statistically significant (*χ*^2^ = 1.88, *p* = 0.17). Interestingly, FTM varies between regions (*χ*^2^ = 75.00, *p* < 2 x 10^−16^; table 2), but not between seasons (*χ*^2^ = 3.83, *p* = 0.15; table 2).

**Table 2.** Summary table of the FTM statistical model. (Results from *χ*^2^ statistics per variable are explained in the text. The reference level for tusk type is ‘short-tusk’, for season is ‘cold’ and for region is ‘East-Katha’. p-value code : 0 ‘***’ 0.001 ‘**’ 0.01 ‘*’)

log(FTM) ~ tusk + age + season + region + (1|ID) + (1| batch)		
random intercept	variance	s.d.
elephant ID	0.0022	0.04673
batch	0.4576	0.67644
residual	0.3138	0.56017

fixed effect	estimate	standard error	*Z* value	Pr (>*Z*)
intercept	4.257	0.208	20.462	<2 × 10^−16^ ***
tusk type (long-tusk)	0.033	0.032	1.039	0.2986
age	0.002	0.001	2.789	0.005 **
season hot	0.064	0.034	1.888	0.06
season monsoon	0.015	0.034	0.436	0.663
region Kawlin	−0.437	0.078	−5.541	3.01 × 10^-8 ***^
region West Katha	0.244	0.051	4.818	1.45 × 10^−6^ ***

### 3.2. Personality

#### 3.2.1. Confirmatory factor analysis

First, we tested whether tusk types differed in personality by comparing the scores along the three main personality factors using CFA per tusk type. Absolute fit indices indicate an acceptable model for long-tusk males but less for short-tusk males (long-tusk: CFI and TLI > 0.95, RMSEA and SRMR = 0.07; short-tusk: CFI and TLI > 0.81; RMSEA = 0.17; table 3). Measurement invariance indicated that both long- and short-tusk males had a three-factor personality construct: attentiveness, sociability and aggressiveness (table 4). We did not find significant differences in the mean factor scores of attentiveness, sociability and aggressiveness between tusk types (table 5), indicating that elephants with different tusk types do not differ in the three personality factors.

**Table 3.** CFA output and estimators for long- and short-tusk males. (CFI, comparative fit index; TLI, Tucker–Lewis index; RMSEA, root mean square error of approximation; SRMR, standardized root mean square residual.)

	d.f.	*χ* ^2^	*p*‐value	CFI	TLI	RMSEA	SRMR
long-tusk	87	131.705	0.001	0.964	0.957	0.07	0.040
short-tusk	87	183.015	0.000	0.841	0.809	0.168	0.069

**Table 4.** Results of invariance test of long- and short-tusk males (long-tusk: *n* = 80; short-tusk: *n* = 25). (Restricted model 1 corresponds to all loadings of items on the factors being the same for long- and short-tusks males; restricted model 2 corresponds to all loadings and intercepts of items on the factors being the same for long- and short-tusks males.)

models	d.f.	*χ* ^2^	*χ*^2^ difference	*p*-values
unrestricted model	174	314.74		
restricted model 1	186	327.22	9.9704	0.6186
restricted model 2	198	336.27	12.6798	0.3927

**Table 5.** Mean factor score differences among long- and short-tusk males for each personality factor.

	estimate	standard error	*Z*-value	*p*(>|*z*|)
attentiveness	0.27	0.34	0.78	0.44
sociability	0.29	0.24	1.20	0.23
aggressiveness	0.34	0.22	1.53	0.13

#### 3.2.2. Exploratory factor analysis

The EFA revealed differences in the descending order of trait loadings in the factor structure, with distinct loadings for each factor between the two tusk types (table 6). For the attentiveness personality factor, the top two loading items (attentive and active) load higher in long-tusk males (loadings: 0.97 and 0.86; table 6), whereas obedient and slow represent strong indicators of the same factor in short-tusk males (loadings: 1.01 and 0.85; table 6). For the sociability personality factor, ‘social’ had the highest loading for both tusk types, with the most significant difference seen in ‘affectionate’, ranking as the third and sixth loading for long- and short-tusk males, respectively (0.72 versus 0.48; table 6). Regarding the aggressiveness personality factor, long-tusk males exhibited a greater association with dominance and a lower association with aggression (0.83 versus 0.80; table 6), while short-tusk males demonstrated an opposite pattern, having more aggressive and less dominant association (0.91 versus 0.72; table 6).

**Table 6.** Structure of trait loadings on personality factors of long-tusk male elephants (*n* = 80 individuals and 109 observations) and of short-tusk male elephants (*n* = 25 individuals and 40 observations) obtained from ‘oblimin’ rotation EFA. (Salient trait loadings ≥0.4, i.e. the factor loadings that meet the criteria for significance to determine the appropriate number of factors to retain in EFA, are marked in bold.)

tusk types	long-tusk males			short-tusk males			
items	attentiveness	sociability	aggressiveness	items	attentiveness	sociability	aggressiveness
attentive	**0.97**	−0.07	−0.01	obedient	**1.01**	0.04	−0.15
active	**0.86**	0.15	−0.12	slow	**0.85**	−0.05	0.00
vigilant	**0.83**	0.02	0.07	confident	**0.79**	0.03	0.18
obedient	**0.82**	0.00	0.12	attentive	**0.74**	0.08	0.16
confident	**0.75**	0.08	0.02	active	**0.70**	0.12	0.15
slow	**0.74**	0.02	0.07	vigilant	**0.59**	0.14	0.30
social	−0.10	**0.91**	0.11	social	−0.11	**0.89**	0.14
mischievous	−0.02	**0.86**	−0.04	playful	0.15	**0.88**	−0.14
affectionate	0.10	**0.74**	0.02	friendly	0.12	**0.75**	−0.08
friendly	0.17	**0.72**	−0.07	mischievous	0.04	**0.74**	0.15
popular	0.05	**0.72**	0.04	popular	0.09	**0.48**	0.42
playful	0.14	**0.68**	−0.01	affectionate	0.38	**0.47**	0.08
dominant	−0.07	0.11	**0.83**	aggressive	−0.04	0.15	**0.91**
aggressive	0.05	0.05	**0.80**	dominant	0.29	−0.14	**0.72**
moody	0.21	−0.13	**0.69**	moody	0.18	0.07	**0.61**

## 4. Discussion

In this study, we investigated differences in FTM levels and personality traits between male Asian elephants with distinct tusk types in Myanmar. To our knowledge, no studies have previously quantified variation in testosterone, personality and tusk length outside musth in Asian elephants. Our findings show no significant differences in testosterone levels between males with different tusk types during non-musth periods. While we did not observe differences in scores along the three main personality factors between tusk types, there were differences in personality structure within these factors: attentive, active and dominant personality items are slightly more pronounced in long-tusk males, whereas short-tusk males display stronger obedient, slower and aggressive association.

Long- and short-tusk males did not differ in average FTM concentrations outside of musth, contrary to our initial hypothesis that long-tusk males would exhibit lower FTM levels. Testosterone concentrations are influenced more by seasonal conditions, geographical factors and food availability [35,56,68,69], and our results support these previous studies. Tusk growth is a complex process involving continuous osteoblastic activity that adds new dentine at the base, gradually pushing the tusk outwards [70,71]. However, the fact that FTM levels are similar between long- and short-tusk males does not exclude a potential hormonal co-regulation on tusk development as ibex and mouflon mechanisms described previously on the androgens relationship. To enhance our understanding of the phenotypic diversity of tusks in Asian elephants, future research could focus more on the metabolic pathways of testosterone and other androgen hormones before tusk emergence and conduct genomic studies to identify candidate genes influencing tusk expression (e.g. [72]).

With regard to personality, we aimed to determine whether males with different tusk types differed in personality traits. CFA indicated no differences in mean factor scores for attentiveness, sociability and aggressiveness between long- and short-tusk males; however, EFA revealed subtle differences in trait loadings within the three personality traits according to tusk type. For attentiveness and sociability, no previous studies have established a connection between tusk types and non-antagonistic personalities, which makes this study one of the first to explore these associations in the Asian elephant. Our results suggest that attentive, active and dominant are more pronounced by long-tusk males than their short-tusk counterparts. Evidence indicates that female Asian elephants prefer larger and, therefore, more dominant males, often responding positively to courtship and allowing extended mounts during mating [26]. These findings are consistent with past observations linking dominance with sexual ornamentation in other species (*Capreolus capreolus,* [21]; *Ovis canadensis*, [73]). Gale [8] noted that in contests between tusked and tuskless males of similar age, size and mass, tusked males typically prevail [8].

Consistent with our expectations, aggressive personality was slightly more pronounced in short-tusk than long-tusk males, but our results indicate that this difference is unlikely to be driven by testosterone. Instead, we believe that the more dominant personality of long-tusk males plays a more important role. Aggression occurs when an elephant causes harm or potentially harms other elephants, including vocalizations, charges, bites or kicks [45]. Aggressive personality among short-tusk males could result from having lower dominance; for example, dominant (long-tusk) elephants may not need to resort to aggression to access resources, while more subordinate (short-tusk) males may need to. The differences in dominance may also explain the active versus slow personality differences between long- and short-tusk males: dominant (long-tusk) elephants can access resources more freely, resulting in an active personality, while subordinate (short-tusk) males may not have access to the same resources or must wait their turn, resulting in a slower or obedient personality. Thus, our findings regarding aggressive, active and slow personalities probably arise from differences in dominance between the tusk types.

Several sources of data could provide useful insights into personality and sexual selection for tusk type. First, personality data in contest and mate choice situations could provide valuable insights into the personality differences between tusk categories that influence female preferences. Second, musth and body size play an important role in the reproductive success of male Asian elephants [22]. Males with different tusk types may differ in musth. For example, when body size and musth period are equal between tusk type, tuskless males are less successful in male-male interactions [22]. Hence, short-tusk males may compensate for the disadvantages of their shorter tusks by evolving a longer or more pronounced musth or a larger body size. It would therefore be informative to quantify whether the musth period or body size differs between tusk types.

In African elephants, the pressure exerted by poaching and the illegal ivory trade has led to genetic shifts in the proportion of individuals possessing tusks [71]. Sukumar, in 1998, developed simulation models to examine potential imbalances in the ratios of tusked versus tuskless males (‘maknas’), as well as shifts in the female-to-male sex ratio [73]. Selective hunting of tusked males has multiple consequences for elephant populations. These include increasingly female-biased sex ratios, erosion of genetic diversity and demographic changes such as reduced mating success among females. Such pressures may also alter social structures and behaviours within the population [74]. If tusk development is governed by genetic mechanisms, then a long-term reduction in long-tusk males could lead to a decline in population heterozygosity [10]. One hypothesis suggests that if females carry the genes for tusk development, the occasional birth of a tusked male could help maintain the frequencies of tusked and tuskless males in the population, thereby buffering the population against the impacts of selective poaching [73]. Additionally, males with larger tusks could have stronger immune systems and lower parasite loads [10], suggesting an association with the immune system, as hypothesized in the immunocompetence handicap hypothesis [74]. Given these associations, investigating the genetic basis of tusk development and its implications for sexual selection and female mate choice is important for preserving phenotypic diversity and the conservation of the Asian elephant.

To conclude, our primary findings indicate significant age-related and regional differences in testosterone levels, but no differences in testosterone levels among males of varying tusk types sampled outside the musth period. This absence of testosterone variation implies that tusk type may be associated with factors beyond testosterone alone, potentially involving genetic and hormonal interactions. Subtle personality differences were noted, with long-tusk males exhibiting more dominant and attentive personality items, whereas short-tusk males tended to be more obedient, slow and aggressive. This study highlights the complex factors associated with tusk differences in Asian elephants. Further investigation into testosterone metabolism, longitudinal studies on tuskless males and comprehensive personality assessments are suggested to enhance our understanding of this poorly understood facet of elephant biology.

## Data Availability

The data and the code can be provided on demand. Supplementary material is available online [75].
